# On biometric systems: electrocardiogram Gaussianity and data synthesis

**DOI:** 10.1186/s13637-017-0056-2

**Published:** 2017-02-21

**Authors:** Wael Louis, Shahad Abdulnour, Sahar Javaher Haghighi, Dimitrios Hatzinakos

**Affiliations:** 1 The Edward S. Rogers Sr. Department of Electrical & Computer Engineering, Faculty of Applied Science and Engineering, University of Toronto, Toronto, Canada; 2 University of Toronto, Toronto, Canada

**Keywords:** Pattern recognition, Electrocardiogram, Data synthesis, Outlier removal

## Abstract

Electrocardiogram is a slow signal to acquire, and it is prone to noise. It can be inconvenient to collect large number of ECG heartbeats in order to train a reliable biometric system; hence, this issue might result in a small sample size phenomenon which occurs when the number of samples is much smaller than the number of observations to model. In this paper, we study ECG heartbeat Gaussianity and we generate synthesized data to increase the number of observations. Data synthesis, in this paper, is based on our hypothesis, which we support, that ECG heartbeats exhibit a multivariate normal distribution; therefore, one can generate ECG heartbeats from such distribution. This distribution is deviated from Gaussianity due to internal and external factors that change ECG morphology such as noise, diet, physical and psychological changes, and other factors, but we attempt to capture the underlying Gaussianity of the heartbeats. When this method was implemented for a biometric system and was examined on the University of Toronto database of 1012 subjects, an equal error rate (EER) of 6.71% was achieved in comparison to 9.35% to the same system but without data synthesis. Dimensionality reduction is widely examined in the problem of small sample size; however, our results suggest that using the proposed data synthesis outperformed several dimensionality reduction techniques by at least 3.21% in EER. With small sample size, classifier instability becomes a bigger issue and we used a parallel classifier scheme to reduce it. Each classifier in the parallel classifier is trained with the same genuine dataset but different imposter datasets. The parallel classifier has reduced predictors’ true acceptance rate instability from 6.52% standard deviation to 1.94% standard deviation.

## Introduction

**Fig. 1:**
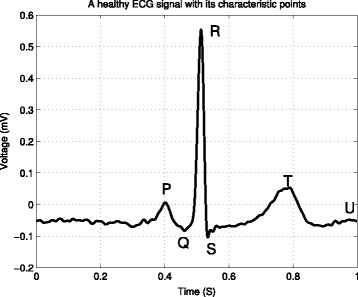
ECG heartbeat with fiducial points

Electrocardiogram (ECG) signal is a quasi-periodic signal with a frequency of 1–1.5 heartbeats per second. It is a recording of the electrical activity in the heart. An ECG signal consists of ECG heartbeats, and each healthy heartbeat has the fiducial points P, Q, R, S, T, and U as illustrated in Fig. 1. Heartbeats have recently been used as a biometric modality. Biometrics is the field of study that models people’s identity using their physical or behavioral traits [1]. After the millennium [2], research concentration on biometrics from signals that are available to all human beings and from signals that are hard to spoof has increased. Some of the biomedical signals that have been used as biometrics are as follows: electromyogram (EMG) [3], muscle signal; phonocardiogram (PCG) [4], heart sound; photoplethysmogram (PPG) [5], organ’s volumetric measure; electroencephalogram (EEG) [6], brain electrical signal; and ECG [7]. Among all these medical signals, the ECG signal is widely used and studied worldwide to diagnose heart problems. Therefore, apart from establishing extensive knowledge about ECG signal by the scientific community, inexpensive sensing devices to acquire the signal have been produced. For this reason, ECG as biometrics can be an inexpensive system to deploy.

Biometric systems require a training stage (interchangeably called enrollment stage) to verify/identify individuals. During the training stage, subjects identities are modeled and stored in a database. Intuitively, the bigger the sample size (the number of observations) the better the model. However, collecting large number of training data can sometimes be troublesome. For example, in forensic applications, one may have few fingerprints or mug shots of a subject to model. Collecting training data can also be expensive and inconvenient. For instance, an ECG signal would require minutes of clean data acquisition to construct a distinctive dataset. Sparing such amount of time might not be feasible. An airport is a fast-paced environment example where requiring minutes to collect data is not preferred.

The most common configuration to set up ECG electrodes is the 12-lead configuration which uses ten electrodes. Six of the ten electrodes are connected to the chest, and four electrodes are connected to the limbs. Misplacing the electrodes affects the acquired ECG signal morphology [8]. Using the 12-lead configuration as a wearable device may not be very attractive due to its inconvenient electrode setup. Other configurations such as a 1-lead configuration [9], which collects ECG signals from fingertips using three electrodes, are more appealing. However, it is more prone to noise than the 12-lead configuration.

In this paper, we tackle the problem of having a small sample size. There are two issues that give rise to this problem. First, the signal is noisy; thus, removing noisy heartbeats reduces the number of observations in the dataset. Also, the ECG heartbeat is a slow signal to acquire especially if compared to other biometrics traits such as video-based face recognition where it is possible to stream 30 frames per second. For practical applications, the extent of people’s patience to cooperate and provide their data for enrollment has been recently studied [10–12].

For this paper, we simulate a small sample size environment by allocating a small number of observations to train a model. We chose an arbitrary number of 20 observations as our baseline since we aimed to have 30–40 s of an enrollment session which we decided on from reports on people’s patience in [10–12]. This enrollment session length can provide a possible range of 20–30 heartbeats. We arrived at this number based on observations from the outlier removal experiment in a subsequent section and our work in [13]. We propose two contributions in this paper: the first is to synthesize ECG heartbeats to increase the number of observations, and second, to study the Gaussianity of ECG signals. Furthermore, due to the small sample size, instability in subjects models occurs; hence, we stabilize the model by fusing several classifiers in a parallel scheme.

This paper is organized such that Section 2 reviews the literature. Section 3 presents the examined database, method of evaluation, and the preprocessing stage along with heartbeat data synthesis and the parallel classifier. Section 4 provides experiments and results. Lastly, Section 5 concludes this paper.

## Literature review

The problem of having a small sample size persists among most biometric systems. Several approaches are available to tackle this problem such as dimensionality reduction, data synthesis, and cascade classifiers which deal with data imbalance.

Most of the work in the literature apply dimensionality reduction techniques. In [14], the authors claimed that when dimensionality reduction is used, the accuracy increases when sample size increases; however, it starts decreasing when a specific sample size is reached. Feature selection and feature extraction are other approaches to handle the small sample size issue, and they are similar in concept to dimensionality reduction. The work in [15] acknowledged the dimensionality reduction problem but claimed that using support vector machine (SVM) can be a viable approach since it generalizes with small sample size and high dimensional space. On the other hand, the work in [16] reported that SVM underperformed when compared to bagging classification in ECG biometrics. In [17], the authors examined several dimensionality reduction techniques with different feature selection methods (Wrapper and ReliefF), feature extraction (principal component analysis (PCA)), and classifiers (K-nearest neighbor, linear discriminative analysis (LDA), Naive Bayes, SVM, and others). It was demonstrated that the highest accuracy was achieved using ReliefF and PCA since they better generalize the data. In [18, 19], the authors proposed quadratic-like discriminative analysis. In this paper, we compare our proposed work to several dimensionality reduction techniques.

Generating synthesized data is mostly examined in face recognition due to symmetry of the face. In [20], the authors generated mirror images of the original image and generated extra left and right symmetrical images. In [21], the authors proposed a cascade classifier where each classifier was trained with a fixed number of samples to reduce data imbalance. Lastly, some techniques are oriented towards synthesizing ECG heartbeats, but they are not for biometrics applications as in [22]. The work in [22] extracts all fiducial points in Fig. 1, and we argue that error in extracting these fiducial points negatively affects the performance of a biometric system.

## Methodology

Verification biometric system is the focus of this paper. A verification biometric system is a binary classification problem to separate two classes: genuine and imposter. The genuine class corresponds to data acquired from the subject that needs to be modeled while the imposter class corresponds to data collected from subjects other than the genuine subject. The imposter class dataset is much larger than the genuine class dataset since any subject that is not genuine can be considered as an imposter. In two-class classification problems, classifiers need to be trained with both genuine and imposter datasets in order to design a function that can separate them. If an imbalanced number of data is used, bias occurs and accuracy is sacrificed. If the number of imposter data is reduced to be in balance with the number of genuine data, the biometric system does not perform too well. Table 1 presents this phenomena.

**Table 1:** Experiment illustrating data imbalance influence on accuracy

# of imp. obs.	20	40	60	80	100	150	200	250
EER (%)	10.41	9.51	9.70	9.77	9.89	9.35	10.00	9.74
TRR (%)	89.59	95.38	97.50	98.21	98.80	99.32	99.56	99.70
TAR (%)	88.93	80.11	71.68	66.04	60.54	51.85	44.76	38.83

We used 20 observations for genuine data. Despite the fact that EER was not influenced greatly when number of imposter data increased, TAR has decreased significantly and TRR has increased. This suggests that the classifier became biased towards imposter data. EER, TAR, and TRR quantities and their calculations are explained in Section 3.2. TRR and TAR are calculated for the 50% decision threshold of selection between imposter and genuine classes *EER* equal error rate, *TAR* true acceptance rate, *TRR* true rejection rate

In this paper, we propose to study the Gaussianity of ECG signal then synthesize it based on a parametric model (Gaussian) to increase sample size. The main point of increasing the sample size is to reduce the imbalance in number between genuine and imposter data. We also use a parallel classifier scheme to reduce instability in classifiers. Before delving into the proposed work, the used database throughout this paper along with the method of evaluation is presented.

### University of Toronto database

Throughout the past century, clinics have collected several ECG databases. However, most of these databases are for medical purposes. In our work, we rely on the University of Toronto database (UofTDB). This database was collected at the University of Toronto [9]. This paper examined 1012 subjects. UofTDB was recorded from fingertips with single lead and with sampling rate of 200 Hz. Each subject has a data recoding of 3 min on average. We used the dataset of 1012 to achieve scalability in low-performance variance.

### Method of evaluation

Quantities and their calculations that are used throughout this paper are explained in this section. False acceptance rate (FAR), false rejection rate (FRR), true acceptance rate (TAR), true rejection rate (TRR), receiver operating characteristic (ROC) curve, and equal error rate (EER) were the main measures used to assess the quality of the proposed system. Each tested dataset has *G*+*I* samples, with *G* being the number of *genuine* heartbeats and *I* being the number of *imposter* ECG heartbeat samples. We define the number of true positive, nTP, as the number of correctly classified genuine heartbeats. Similarly, the number of true negative, nTN, is defined as the number of correctly classified imposter heartbeats. Moreover, the number of false positive, nFP, is the number of misclassified imposter heartbeats as genuine heartbeats. Likewise, the number of false negative, nFN, is the number of misclassified genuine heartbeats as imposter heartbeats. Following these definitions:

1 $$ \text{FAR}=\frac{\text{nFP}}{I}, \text{FRR}=1-\frac{\text{nTP}}{G}  $$

Also TRR=1−FAR and TAR=1−FRR. ROC curves measure the performance of a system in different operating points. An ROC curve plots FRR versus FAR. Closely related is EER. EER is the error on the operating point for which FAR is equal to FRR.

### Preprocessing

ECG signal is one among other human body-generated electrical signals. Other electrical and non-electrical signals may interfere with ECG signal acquisition (e.g., EMG signal). Respiration also interferes with the acquisition on the range of frequencies of 0.15–0.30 Hz [23]. External environment signals such as contact noise, power-line interference (50 or 60 Hz), and electrode movements (1–10 Hz) are other sources of noise. A fourth-order band-pass Butterworth filter with cutoff frequencies of 0.5–40 Hz was applied to the signal as a first stage of preprocessing. Afterwards, ECG signals were isolated into heartbeats and were centered at the R peaks with 500-ms duration from each side of the peak [16]. R peaks were detected using Pan-Tompkins [24].

After segmenting the signal, we removed outliers using the Gaussian mixture model (GMM) online outlier removal in [13]. If we model *normal* heartbeats, then any heartbeat with statistics significantly different from the *normal* heartbeat model is classified as an *abnormal* heartbeat. Hence, we constructed a *normal* heartbeat model. For the task, *normal* heartbeat segments were collected to train the GMM. We used the GMM as a one-class classifier unlike the usual work in the literature which uses it as an unsupervised clustering method. GMM is a sum of *M*-weighted Gaussian densities [25] given by

2 $$ P(\boldsymbol{x})=\sum_{m}^{M}w_{m}p(\boldsymbol{x},\mu_{m},C_{m})  $$

where *w*_*m*_ are the weights of the Gaussian densities, $\sum _{m}^{M} w_{m}=1$. ***x*** is a *k* dimensional feature vector. Therefore, the probability density function, *p*(***x***,*μ*_*m*_,*C*_*m*_), is

3 $$ {{}\begin{aligned} p(\boldsymbol{x},\mu_{m},C_{m})=\frac{1}{(2\pi)^{\frac{k}{2}}(|C_{m}|)^{\frac{1}{2}}}e^{-\frac{1}{2} (\boldsymbol{x}-\mu_{m})^{T}C_{m}^{-1}(\boldsymbol{x}-\mu_{m})} \end{aligned}}  $$

where *μ*_*m*_ and *C*_*m*_ are the mean vector and the covariance matrix, respectively. Also, |*C*_*m*_| is the determinant of the covariance matrix.

If we have a vector of 200 features (i.e., *k*=200), then each Gaussian distribution is of 200 dimensions. The motivation behind using the GMM was the assumption that *normal* ECG heartbeats could be modeled into *M* Gaussian densities, each in *k* dimensions.

The expectation maximization (EM) [26] algorithm was used to construct the GMM. EMconsiders all training examples and attempts to fit a Gaussian distribution on it. The training steps would be as the following:

1. Compute the probability that the training sample ***x*** belongs to the Gaussian *m* using
$P(\boldsymbol {x}|m)=\frac {w_{m}^{(i)}p(\boldsymbol {x},\mu _{m}^{(i)},C_{m}^{(i)})} {\sum _{j}^{M}w_{j}^{(i)}p(\boldsymbol {x},\mu _{m}^{(i)},C_{m}^{(i)})}$,where $P(\boldsymbol {x}|\mu _{m}^{(i)},C_{m}^{(i)})$ is used to indicate that these values depend on the previous iteration
2. Estimate the new weight $w_{m}^{(i+1)}=\frac {1}{T}\sum _{t=1}^{T}P(\boldsymbol {x}_{\boldsymbol {t}}|m)$
3. Estimate the new mean $\mu _{m}^{(i+1)}=\frac {\sum _{t=1}^{T}P(\boldsymbol {x}_{\boldsymbol {t}}|m)\boldsymbol {x}_{\boldsymbol {t}}} {\sum _{t=1}^{T}P(\boldsymbol {x}_{\boldsymbol {t}}|m)^{2}}$
4. Update the variance $\sigma ^{2(i+1)}_{m}=\frac {\sum _{t=1}^{T}P(\boldsymbol {x}_{\boldsymbol {t}}|m)\boldsymbol {x}_{\boldsymbol {t}}} {\sum _{t=1}^{T}P(\boldsymbol {x}_{\boldsymbol {t}}|m)}-\mu ^{2}_{m}$

where *T* is the number of observations in the training dataset. There is no specific method for termination; however, it is usually based on a heuristic approach.

#### Evaluation procedure

After obtaining the Gaussian models from the training data, the evaluation was based on the log-likelihood measurement. Log-likelihood measures quantitatively the likelihood that the tested data belong to the mixture. Choosing the minimum negative log-likelihood is equivalent to choosing the maximum likelihood.

**Fig. 2:**
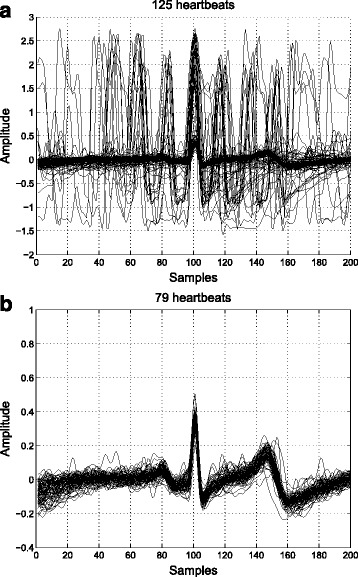
GMM, *M*=2 model outlier removal. **a** Before outlier removal; **b** after applying GMM, *M*=2 outlier removal

GMM with two components (GMM, *M* = 2) was trained on a dataset of *normal* heartbeats. GMM, *M*=2 was used in particular due to our previous work results in [13]. The collection of *normal* heartbeats was conducted by removing *abnormal* ECG heartbeats from the examined pool of heartbeats. A heartbeat that was significantly different from healthy ECG morphology which contains P, Q, R, S, T, and U fiducial points was considered as an *abnormal* heartbeat. In other words, the R peak of the heartbeats were first detected by Pan-Tompkins algorithm, then these heartbeats were manually inspected to ensure they follow the morphology in Fig. 1 to decide whether they are *normal* or *abnormal* heartbeats. During biometric system experiments, every heartbeat in the examined database was passed through this outlier removal to measure heartbeat quality and to decide whether to keep (i.e., classify as *normal*) or to eliminate (i.e., classify as *abnormal*). Figure 2 demonstrates ECG signal heartbeats before and after outlier removal. Table 2 presents the EER for the biometric system with and without outlier removal, and it also reports the number of observations examined. It can be noticed that almost half the observations were removed by applying this method of outlier removal. Other outlier removal approaches might be used, but the GMM-based outlier removal is an online outlier removal that depends on current and previous observations only, and it is subject invariant. Hence, it is more desirable in practical applications. Therefore, it was used in the paper. Despite the achieved high accuracy, around 50% of the heartbeats were classified as *abnormal* heartbeats; consequently, using such outlier removal may give rise to the issue of small sample size. Also, for this reason, having 30–40 s of enrollment means we would collect an average of 20 clean observations, which was used as a baseline in this paper.

**Table 2:** Biometric system performance with outlier removal system without limiting training sample to 20 observations

Method	EER (%)	No. of observations
No outlier removal	9.44	158,984
GMM, *M*=2	5.94	78,655

### ECG heartbeats synthesis

We hypothesize that ECG heartbeats exhibit a multivariate Gaussian distribution. However, the influence of internal and external factors deviate the model from Gaussianity. We attempt to capture this underlying Gaussianity. Each observation consisted of 200 time samples (random variables) since the sampling rate is 200 Hz, and we segmented the heartbeats to have a 1-s duration. As mentioned earlier and as shown in Table 1, we desired to generate data that can be appended to the genuine dataset to reduce data imbalance and to reduce bias towards imposter dataset.

We modeled the genuine data $\mathbf {X}\in \mathbb {R}^{n\times k}$, where *n* is the number of observations and *k*=200 is the number of dimensions. Therefore, an observation **x** with *k* dimensions has probability density $p(\mathbf {x})\sim \mathcal {N}(\mu,\boldsymbol {\Sigma })$ such that:

4 $$ p(\mathbf{x})=\frac{1}{(2\pi)^{k/2}|\boldsymbol{\Sigma}|^{1/2}}e^{-\frac{1}{2}(\mathrm{x}-\mu)^{t} \boldsymbol{\Sigma}^{-1}(\mathrm{x}-\mu)}  $$

where $\boldsymbol {\mu }\in \mathbb {R}^{k}$ is the mean of **X**, ***Σ*** is the covariance matrix of **X**,|***Σ***| is the determinant of the covariance matrix, and ***Σ*** ^−1^ is the inverse of the covariance matrix. A synthesized observation is generated by drawing a random vector from this distribution.

**Fig. 3:**
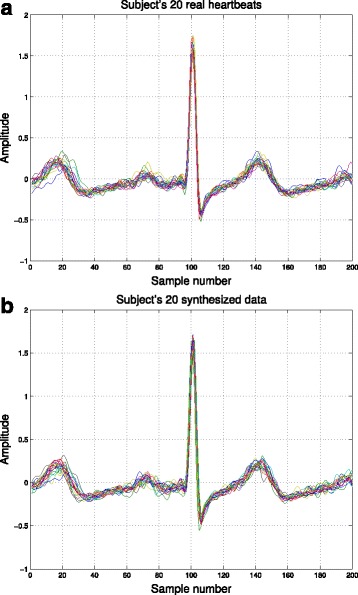
Synthesized data generation from multivariate Gaussian distribution. **a** Real heartbeats; **b** synthesized heartbeats

A set of data synthesis is in Fig. 3. This result was not surprising. Prior to making such multivariate hypothesis, we analyzed the Gaussianity of the ECG heartbeat. We used Royston’s test [27, 28] for multivariate normality test. It is based on Shapiro-Wilk’s test [29], a univariate normality test. Royston’s test checks normality of each variable alone using Shapiro-Wilk’s test, then it combines Shapiro-Wilk statistics into one statistics test for multivariate distribution. The combined multiple statistics would approximate a *χ* ^2^ random variable when the data is a multivariate Gaussian distribution. If *W*_*j*_ is Shapiro-Wilk’s test of the *j*th variable in the multivariate data, then Royston’s test, *R* [30, 31]:

5 $$ R_{j}= \left[ \phi^{-1} \left(\frac{1}{2} \phi\left(-\frac{{(1 - W_{j})}^{g} - m}{s}\right)\right) \right]^{2}  $$

Parameters *g*,*m*,and*s* are calculated from polynomial approximation. *ϕ*(.),*ϕ* ^−1^(.) are the CDF and its inverse for the Gaussian distribution, respectively. If we have *p* variates, then the aggregation of *R*_*j*_ in Eq. 6 would have a *χ* ^2^ distribution.

6 $$ H=e\sum_{j=1}^{p} \frac{R_{j}}{p}  $$

*e* is the equivalent degree of freedom and is calculated as:

7 $$ e=\frac{p}{1+ (p-1)C}  $$

where *C* is calculated as the average of the correlations of *R*_*j*_s. Furthermore, we utilized Sequential Forward Selection (SFS) [32] algorithm with Royston’s test on the training dataset to investigate the number of variables that constitutes a multivariate normal distribution. The algorithm we implemented for multivariate Gaussian analysis is in Algorithm 1. This algorithm incorporates SFS with Shapiro-Wilk’s and Royston’s tests.



After running Algorithm 1, ECG heartbeats could successfully have multivariate normality with more than 20 variables out of the 200 variables. In other words, around 20 out of 200 dimensions could constitute a multivariate normal distribution. This multivariate Gaussianity helps us capture the underlying Gaussianity of the heartbeats and supports our hypothesis that it is most likely that ECG heartbeats exhibit a multivariate Gaussian distribution if there are no changing factors that affect its morphology. Also, experiments based on such assumption improved biometric system performance.

In other words, we assume that ECG heartbeats for each individual exhibit a multivariate Gaussian distribution; nevertheless, the changes in ECG heartbeat morphology due to diet, physical and psychological changes, and other factors deviate the signal from Gaussianity. From this Gaussian model, we create the synthesized ECG heartbeats.

### Parallel classifier to reduce instability

**Fig. 4:**
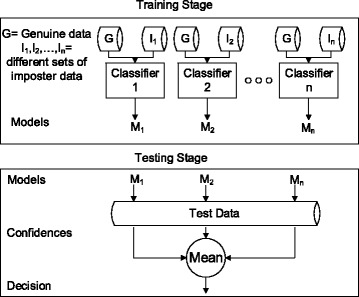
Parallel classifier scheme

The main purpose of data generation is to increase biometric system performance by making use of the abundance of imposter dataset. The number of real genuine observations is small; we restricted it to 20 observations. On the other hand, we have thousands of imposter data. Due to small number of real genuine observations, classifiers’ structures change significantly depending on the imposter data that train the classifiers. We propose to use a parallel classifier structure, and Fig. 4 presents the scheme for it. All classifiers within the parallel classifier were trained with same set of genuine training dataset, but each classifier was trained with a different set of imposter data. The *mean* value of confidences of the classifiers’ outputs was used to make a classification decision.

## Experimentation

This section investigates three main experiments: first, it presents biometric system improvement as a result of data synthesis; second, the experiment compares biometric system accuracy with data synthesis versus systems with different dimensionality reduction techniques from the literature; and lastly, the third experiment demonstrates the parallel classifier performance. Throughout all experiments, the bagging classifier was used.

There are several classification methods in the literature, and bagging [33] is one of them. In a nutshell, bagging is a machine learning technique that generates predictors on merely re-sampled data. The aggregated average of predictors makes a decision. Bagging was used in particular because we observed an unstable classifier prediction when we examined ECG heartbeat data. It was unstable in a sense that a slight change in the training data led to a significant change in the construction of the classifier and a significant change in accuracy. Bagging usually reduces this issue [33]. Work in [34] suggests the superiority of bagging over other classifiers.

Suppose a training dataset, $\mathcal {L}$, is populated with data {*y*_*n*_,**x**_**n**_,*n*=1,…,*N*}, where *y* is the data class and **x** is the input data. From these samples, bagging generates multiple bootstrap samples, $\mathcal {L}^{(B)}$, from $\mathcal {L}$. For each $\mathcal {L}^{(B)}$, it finds a predictor that predicts the class, *y*. Bootstrapping samples, $\mathcal {L}^{(B)}$, are constructed by drawing *N* samples with replacement from $\mathcal {L}$. The predictor used with bagging in this paper is the simple decision tree. The final decision on the class is made by voting.

### Synthesized ECG heartbeat generation

This experiment reports the improvement achieved in a biometric system’s EER, TAR, and TRR quantities. Synthetic data were generated as explained in Section 3.4. The generated data were added to the pool of real genuine data, and they were used to train a bagging classifier. Table 3 presents an experiment when the real genuine data were restricted to 20 observations for every subject.

**Table 3:** Experiment for 20 real genuine data

Number of synthesis	Number of imposter samples							
	20	40	60	80	100	150	200	250
EER (%)								
0	10.41	9.51	9.70	9.77	9.89	9.35	10.00	9.74
50	10.07	8.59	8.37	7.46	7.55	7.69	7.78	7.48
100	9.84	8.64	7.71	7.41	7.46	7.50	7.59	7.34
200	9.51	8.75	7.98	7.60	7.64	7.46	*6.71*	7.18
400	9.80	8.53	8.16	7.82	7.76	7.44	7.36	7.06
TAR (%)								
0	88.93	80.11	71.68	66.04	60.54	51.85	44.76	38.83
50	93.99	89.51	85.36	82.90	80.43	74.91	69.88	66.55
100	94.58	90.54	88.04	85.75	83.78	79.79	76.20	73.08
200	95.37	91.54	89.06	87.02	85.48	82.22	79.53	76.99
400	95.51	92.12	90.00	88.61	87.21	84.37	81.85	80.08
TRR (%)								
0	89.59	95.37	97.50	98.21	98.80	99.28	99.56	99.70
50	85.45	92.97	95.68	96.63	97.43	98.40	98.89	99.15
100	84.82	92.11	94.83	96.00	96.82	97.95	98.51	98.83
200	83.24	91.25	93.90	95.19	96.09	97.36	98.02	98.43
400	82.28	90.06	93.03	94.34	95.32	96.71	97.45	97.91

Synthetic genuine data are appended with real genuine data in training the bagging classifier

From Table 3, it can be noticed that the best EER from the examined experiments was achieved when we trained a classifier with 220 genuine observations (200 synthesized genuine data + 20 real genuine data) and 200 imposter data. Hence, this proves that adding data synthesis improves results. One may inquire why do we not consider the TAR of 400 synthesis data and 20 imposter data as the best result? The reason is TAR, unlike EER which considers both TAR and TRR, ignores TRR. TRR for the same experiment (400 synthesized data and 20 imposter data) has a significant drop from the average TRR of all experiments; it has a TRR 82.28*%*. The reason behind that is that the classifier is biased towards the genuine data. It is worth mentioning that the reported TAR and TRR were calculated when the operating threshold that splits genuine from imposter classes for the bagging classifier was assigned to 50%.

From Table 3, a trend can be noticed that increasing the number of synthesized samples does indeed improve the result. However, it can improve the result to the extent where real genuine data start to get concealed by the abundance of the synthesized data. From this point onwards, the model turns to be mostly a multivariate Gaussian distribution only, i.e., it can be described by mean and standard deviation parameters. This model by itself might not be descriptive enough to classify a large number of subjects adequately, e.g., the 1012-subject database in UofTDB.

We considered a baseline of 20 real genuine observations, but we also conducted other experiments when real genuine dataset has 30 and 60 observations. Table 4 tabulates the EER that was achieved along with its corresponding number of synthesized data and number of imposter data. This table further confirms our hypothesis on the fact that adding the proposed generated synthesized data reduces data imbalance and constructs a better classifier.

**Table 4:** Experiment for 30 (top) and 60 (bottom) real genuine observations to: emphasize on performance improvement when we have 30 and 60 genuine observations instead of 20 genuine observations (Table 3), show that data synthesis improves the results, and lastly, to observe the upward improvement when training sample size was increased using data drawn from Gaussian distribution

Number of synthesis	Number of imposter samples							
EER (%)	20	40	60	80	100	150	200	250
Training with 30 real genuine dataset								
0	9.06	8.24	7.96	7.87	8.19	8.44	7.56	8.40
50	8.71	7.88	7.71	7.47	6.77	6.68	6.68	7.25
100	9.05	8.04	7.16	6.77	6.88	6.77	6.60	6.27
200	8.49	7.77	7.27	7.06	7.05	6.21	6.66	6.33
400	9.06	7.99	7.76	7.41	7.33	6.99	6.15	6.38
Training with 60 real genuine dataset								
0	7.38	6.50	6.36	6.01	6.06	5.98	5.82	5.38
50	7.24	6.39	6.17	5.98	5.37	5.12	5.63	5.17
100	7.35	6.35	6.20	5.97	5.46	5.17	5.03	5.13
200	7.54	6.52	5.91	5.70	5.56	5.28	5.08	5.28
400	7.35	6.43	6.34	6.07	6.02	5.64	5.40	5.04

### Comparison to dimensionality reduction

**Fig. 5:**
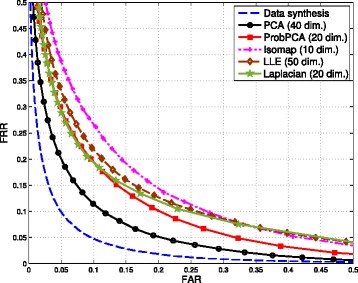
ROC curves for the biometric system with different dimensionality reduction techniques

Dimensionality reduction is one of the most used techniques in the literature to deal with the small sample size problem [14, 15]. In this experiment, we compared the biometric system with data synthesis versus biometric systems with PCA, probabilistic PCA [35], Isomap [36], Laplacian [37], and local linear embedding (LLE) [38]. In all of these biometric systems, real genuine data of 20 observations were used and a wide range of numbers of imposter data and numbers of reduced dimensions were experimented. Table 5 tabulates the results with the examined parameters that achieved the lowest EER while Fig. 5 computes ROC curves for the biometric systems with data synthesis and all dimensionality reduction techniques with parameters that achieved the lowest EER. It is pertinent to mention that all biometric systems were implemented in an identical environment using same sets of real genuine and imposter observations.

**Table 5:** Dimensionality reduction techniques with parameters that achieved the lowest EER

Method	EER (%)	No. of impos.	No. of dim.
PCA	9.92	20	40
Prob. PCA	13.47	120	20
Isomap	16.16	120	10
LLE	14.82	20	50
Laplacian	13.43	250	20
Data synthesis	*6.71*	200	200

### Parallel classifier

Bagging classifier has been investigated for ECG heartbeats due to its capability to reduce instability in predictors. Despite the reduction in instability, some instability still exist. This instability can especially be noticed on performance of individual subjects rather than considering hundreds of subjects when calculating biometric system performance using confusion matrix. Our proposed parallel classifier further reduces such instability by implementing bagging classifiers in a parallel scheme. Table 6 reports the instability result and presents the influence of the parallel classifier in stabilizing it. It can be observed from Table 6 that when there is no parallel classifier, TAR would have a standard deviation of 6.52*%* per subject and TRR of 0.61*%* per subject while when parallel classifier was used, TAR would have a standard deviation of 1.94*%* per subject and TRR of 0.10*%* per subject. The only difference among classifiers in the parallel classifier is that the imposter datasets are different in each classifier. Complexity can be an issue. If training a classifier takes *t* seconds, then training *n* parallel classifiers needs *n*×*t* seconds.

**Table 6:** Standard deviation of TAR and TRR for biometric systems with and without parallel classifier

No. of parallel	TAR standard	TRR standard
classifiers	deviation (%)	deviation (%)
0	±6.52	±0.61
5	±3.63	±0.24
10	±2.43	±0.17
20	±1.94	±0.10

One might wonder that the parallel classifier might make the bagging classifier a redundant stage since both classifiers attempt to do the same task—the aggregate decision of different classifiers trained with different data. Nevertheless, the main difference is that in bagging, we re-sample the data from the same pool while in the parallel classifier, we change the imposter data completely in each classifier. We have conducted an experiment to explain that parallel classifier and bagging complement each other rather than making one as redundant. The experiment was conducted on the highest achieving results in Table 3 (i.e., 20 real genuine with 200 imposter samples and 200 synthesized data). We once created 50 parallel classifiers while using just one decision tree (i.e., no bagging), and once again, we experimented one parallel classifier and bagging with 50 decision trees. Table 7 presents the results.

**Table 7:** Experiment shows that parallel classifier and bagging complement each other

Classifiers	EER (%)
50 parallel classifiers with 1 decision tree (no bagging)	20.98
1 parallel classifier with 50 decision trees (bagging)	6.71

From Table 7, we can conclude that parallel classifier alone does not improve the results greatly or makes bagging redundant but it increases robustness towards changes in the imposter data as noted in Table 6.

## Conclusions

Two contributions have been proposed in this paper: analyzing the Gaussianity of ECG observations and a proper and simple technique to generate ECG heartbeat synthesis. Also a methodology to reduce classifiers’ instability was presented and used. We used Sequential Forward Selection along with Shapiro-Wilk’s univariate and Royston’s multivariate normality tests to find a subset of ECG heartbeat variables that exhibit multivariate normal distribution. Our analysis suggests that more than 20 variables in the ECG heartbeats have multivariate normal distribution. Those multivariate variables capture the main features of ECG heartbeats. Therefore, they assist us in capturing the underlying Gaussianity of heartbeats and further support our hypothesis that ECG heartbeats exhibit a multivariate Gaussian distribution should deviating factors not occur. ECG heartbeat synthesis was used to generate genuine subject data to increase its sample size in a verification biometric system. When only 20 real genuine heartbeats were used and 200 synthesized heartbeats were generated, the biometric system achieved an equal error rate (EER) of 6.71% in comparison to a minimum of 9.35% when data synthesis was not utilized. A biometric system with data synthesis outperformed several other biometric systems which employed dimensionality reduction techniques. The EER of the biometric system with data synthesis outperformed PCA by 3.21%, probabilistic PCA by 6.76%, Isomap by 9.45%, local linear embedding by 8.11%, and Laplacian by 6.72%.

Classifier instability is problematic especially when the sample size of the data is small. Bagging is usually used to reduce such effect, so we used it; however, to further reduce instability, we proposed to use a parallel classifier scheme. All classifiers were trained with the same set of genuine data while each classier was trained with a different set of imposter data. This method reduced classifier instability. Through this scheme, we could reduce the true acceptance rate instability from 6.52*%* standard deviation to 1.94*%* standard deviation. The proposed contributions are expected to produce promising results in other applications.

Currently, we exploited the Gaussianity of ECG heartbeats; nevertheless, other approaches such as deep learning to generate data can be researched in the future. Our preliminary results with deep learning achieve promising results. Furthermore, the maximum number of synthesized data before they start concealing the real genuine data might be set up as an optimization problem, and this is also left as a future work.
